# Unraveling tumour microenvironment heterogeneity in nasopharyngeal carcinoma identifies biologically distinct immune subtypes predicting prognosis and immunotherapy responses

**DOI:** 10.1186/s12943-020-01292-5

**Published:** 2021-01-11

**Authors:** Yu-Pei Chen, Jia-Wei Lv, Yan-Ping Mao, Xiao-Min Li, Jun-Yan Li, Ya-Qin Wang, Cheng Xu, Ying-Qin Li, Qing-Mei He, Xiao-Jing Yang, Yuan Lei, Jia-Yi Shen, Ling-Long Tang, Lei Chen, Guan-Qun Zhou, Wen-Fei Li, Xiao-Jing Du, Rui Guo, Xu Liu, Yuan Zhang, Jing Zeng, Jing-Ping Yun, Ying Sun, Na Liu, Jun Ma

**Affiliations:** 1 Department of Radiation Oncology, Sun Yat-sen University Cancer Center, State Key Laboratory of Oncology in South China, Collaborative Innovation Center for Cancer Medicine, Guangdong Key Laboratory of Nasopharyngeal Carcinoma Diagnosis and Therapy, Guangzhou, 510060 People’s Republic of China; 2 Department of Pathology, Sun Yat-sen University Cancer Center, State Key Laboratory of Oncology in South China, Collaborative Innovation Center for Cancer Medicine, Guangdong Key Laboratory of Nasopharyngeal Carcinoma Diagnosis and Therapy, Guangzhou, 510060 People’s Republic of China

**Keywords:** Nasopharyngeal carcinoma, Tumour microenvironment, Gene expression profiles, Virtual microdissection, Prognosis, Immunotherapy responses

## Abstract

Currently, there is no strong evidence of the well-established biomarkers for immune checkpoint inhibitors (ICIs) in nasopharyngeal carcinoma (NPC). Here, we aimed to reveal the heterogeneity of tumour microenvironment (TME) through virtual microdissection of gene expression profiles. An immune-enriched subtype was identified in 38% (43/113) of patients, which was characterized by significant enrichment of immune cells or immune responses. The remaining patients were therefore classified as a non-Immune Subtype (non-IS), which exhibited highly proliferative features. Then we identified a tumour immune evasion state within the immune-enriched subtype (18/43, 42%), in which high expression of exclusion- and dysfunction-related signatures was observed. These subgroups were designated the Evaded and Active Immune Subtype (E-IS and A-IS), respectively. We further demonstrated that A-IS predicted favourable survival and improved ICI response as compared to E-IS and non-IS. In summary, this study introduces the novel immune subtypes and demonstrates their feasibility in tailoring immunotherapeutic strategies.

**Supplementary Information:**

The online version contains supplementary material available at 10.1186/s12943-020-01292-5.

## Backgroud

Nasopharyngeal carcinoma (NPC) is a heterogeneous epithelial tumour highly prevalent in East and Southeast Asia [1]. It is characterised by Epstein–Barr virus (EBV) infection and heavy lymphocyte infiltration [2]. These special features of the NPC tumour microenvironment (TME) indicate the potential benefits of immune checkpoint inhibitors (ICIs). Unfortunately, the anti-programmed cell death protein 1 (PD-1) therapies benefit only a subset of patients, while there is no strong evidence of the well-established ICI biomarkers in NPC [3]. Tumour gene expression profiles represent important resources to model the TME status and identify potential biomarkers [4, 5]. In this study, we unraveled the TME heterogeneity based on gene expression profiles, and identified the distinct Active, Evaded and non-Immune Subtypes (A-IS, E-IS and non-IS) in NPC (Additional file 1: Figure S1). We further demonstrated their predictive capability for forecasting prognosis and ICI response, thereby providing a strong tool for tailoring immunotherapeutic strategies.

## Results and discussions

### Identification of three distinct NPC immune subtypes

**Fig. 1:**
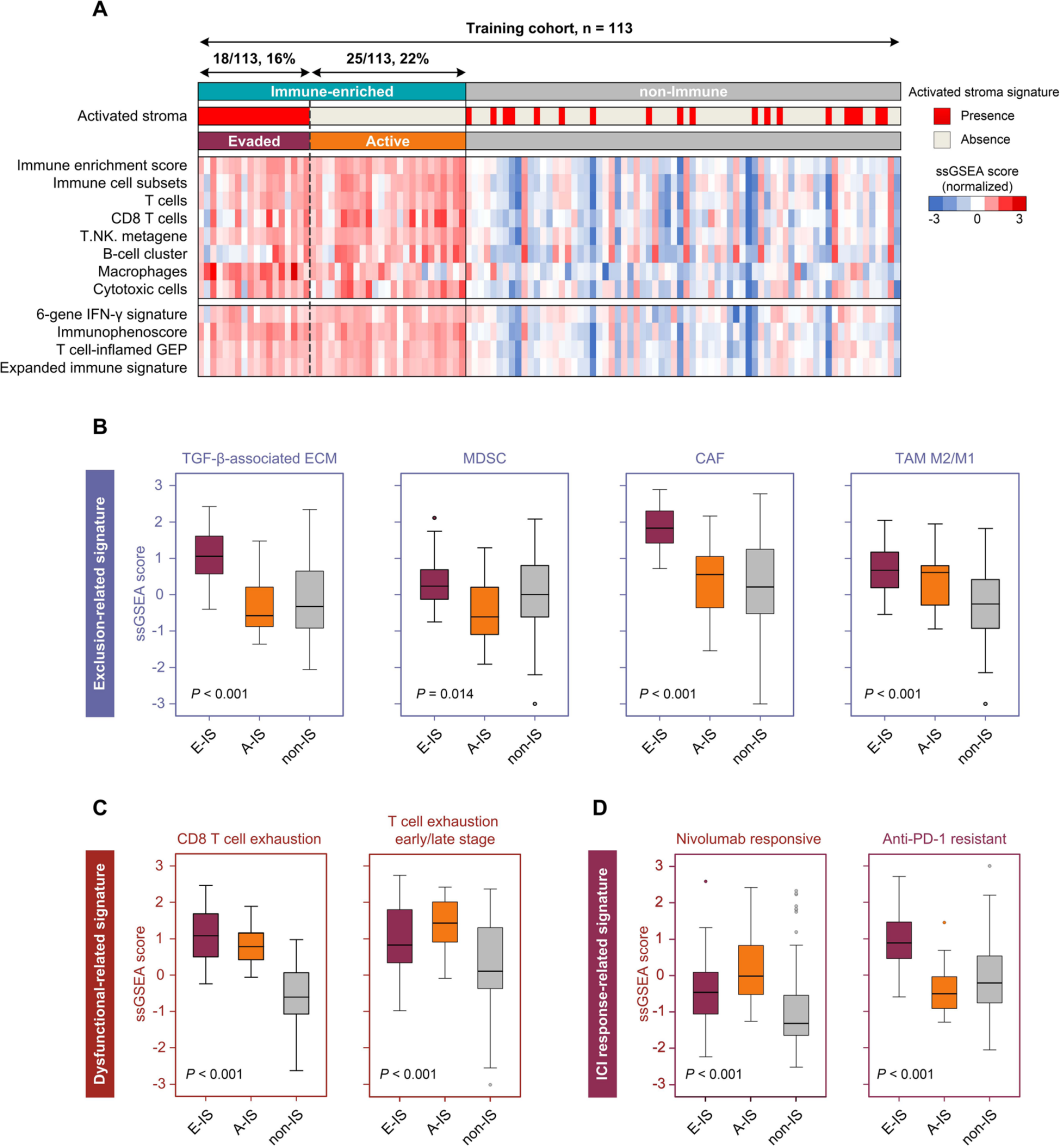
Identification of three distinct tumour microenvironment-based immune subtypes. **a** An immune-enriched subtype (43/113, 38%; green bar) and a non-Immune Subtype (non-IS) (70/113, 62%; grey bar) were identified using an NMF algorithm in the training cohort. The immune-enriched subtype was further subdivided into Evaded (18/113, 16%; purple bar) and Active (25/113, 22%; orange bar) Immune Subtypes (E-IS and A-IS, respectively), using nearest template prediction (NTP) analysis with a signature identifying the activated stroma response. In the heatmap, high and low ssGSEA scores are represented in red and blue, respectively. The presence of an activated stroma signature is indicated in red and its absence is in grey. **b-d** Box plots showing expression of exclusion-related (**b**), dysfunctional-related (**c**), and ICI response-related (**d**) signatures between E-IS, A-IS, and non-IS. The box plot centre corresponds to the median, with the box and whiskers corresponding to the interquartile range and 1.5 × interquartile range, respectively. *P*-values were based on the Kruskal–Wallis rank-sum test. CAF, cancer-associated fibroblast; ECM, extracellular matrix; GEP, gene expression profile; ICI, immune checkpoint inhibitor; MDSC, myeloid-derived suppressor cell; NMF, non-negative matrix factorization; NPC, nasopharyngeal carcinoma; ssGSEA, single-sample gene set enrichment analysis; TAM, tumour-associated macrophage

The non-negative matrix factorization (NMF) approach could microdissect the gene expression patterns of different TME components virtually, and is well suited for biological data that constrains all sources to be positive in nature [6–8] (Additional file 1: Methods). We first applied the NMF algorithm to extract an immune factor (or expression pattern) in 113 NPC samples from the training cohort (Additional file 1: Figure S1–2 and Tables S1–2), and revealed an immune-enriched subtype present in 38% of the cohort (43/113), and a non-IS in the rest (Fig. 1a). Patients with the immune-enriched subtype showed significant enrichment of signatures identifying immune cells or immune response (all, *P <* 0.001). Furthermore, upregulated immunological pathways were observed in the immune-enriched subtype versus the non-IS (Additional file 2: Table S3).

As stromal cells play an important role in modelling tumour immune evasion even in the presence of abundant immune cells [9], we further dissected the gene expression profiles of the patients with the immune-enriched subtype, in which 42% (18/43) was characterized with the presence of a signature identifying an activated stromal response (Fig. 1a) [7]. Exclusion-related signatures, such as TGF-β-associated extracellular matrix (ECM), were highly expressed in those with activated stroma (Fig. 1b). Intriguingly, we observed relatively higher expression of the CD8 T cell exhaustion signature in the immune-enriched subtype compared with the non-IS (Fig. 1c). This reflected an activation-dependent exhaustion expression program in NPC [2]. Of note, the immune-enriched subtype lacking the activated stroma was significantly associated with early-stage dysfunctional T cells (Fig. 1c), suggesting its plastic and therapeutically reprogrammable state. Therefore, we defined the subgroups with or without activated stroma within the immune-enriched subtype as an E-IS and an A-IS, respectively; the antitumour immunity was dampened even with a pre-existing abundance of immune cells in E-IS. Interestingly, higher and lower expression of the nivolumab responsive and anti-PD-1 resistant signatures respectively, were observed in A-IS (Fig. 1d). GSEA revealed an enrichment of pathways, such as hypoxia-response, epithelial mesenchymal transition, and angiogenesis in the E-IS versus A-IS (Additional file 2: Table S4).

### Associations of immune subtypes with tumoural genomic features and prognosis

Interestingly, there were no differences in the tumour mutation burden and copy number alterations between the subtypes (Additional file 1: Figure S3A,B), suggesting that other mechanisms might drive their biological differences. Non-IS was associated with higher scores for cell cycling signature [2] (*P <* 0.001) (Additional file 1: Figure S3C). Assessment of the crucial genetic changes in NPC [1] among the immune subtypes revealed a significantly higher proportion of deletions of *CDKN2A*, a tumour suppressor-related to the cell cycle, in non-IS (30% vs. 5%, *P =* 0.004) (Additional file 1: Figure S3D). These results indicated a proliferative and aggressive status in non-IS. Finally, we explored the prognostic implications of the immune subtypes in 88 patients with available survival outcomes from the training cohort. Patients within A-IS showed a tendency for better progression-free survival (PFS) than those with E-IS and non-IS (*P =* 0.18) (Additional file 1: Figure S3E); the lack of statistical significance may be due to the relatively small sample size in each group.

### Validation of the immune subtypes in four cohorts

**Fig. 2:**
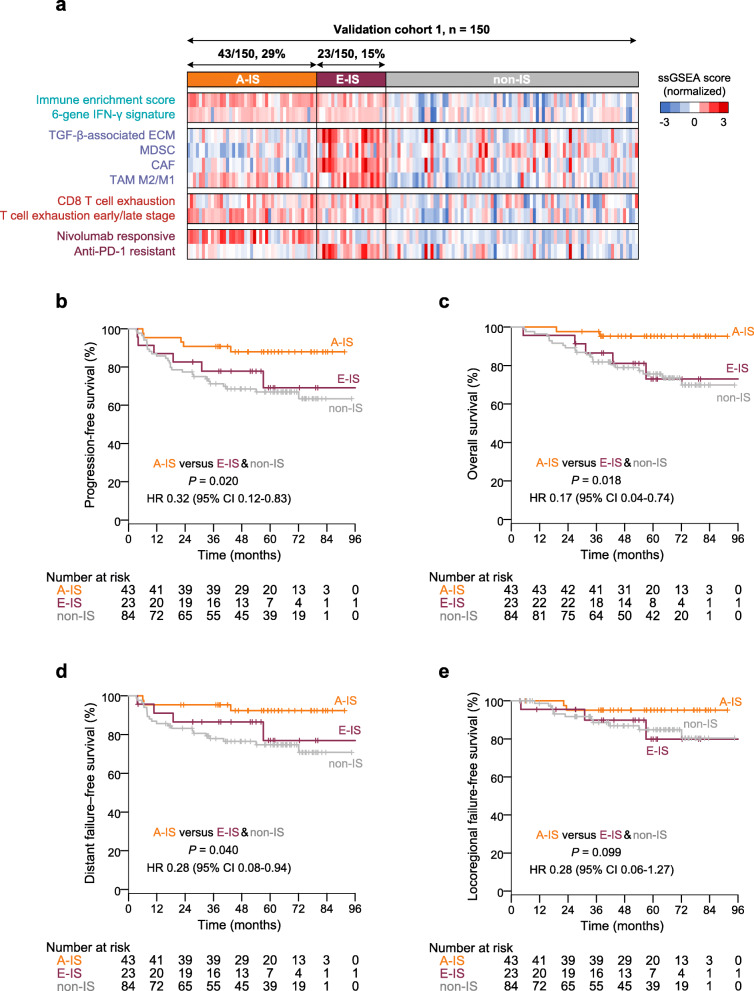
Verification of the immune subtypes in validation cohort 1. **a** Heatmap representation of the expression of immune-related signatures between A-IS, E-IS, and non-IS in validation cohort 1 (*n* = 150). In the heatmap, high and low ssGSEA scores are represented in red and blue, respectively. The presence and molecular characteristics of the immune subtypes were successfully validated. **b**–**e** Kaplan–Meier curves for progression-free survival (**b**), overall survival (**c**), distant failure-free survival (**d**), and locoregional failure-free survival (**e**) according to immune subtypes in validation cohort 1 (*n* = 150). Cox regression HRs and 95% CIs obtained after correcting for age (>45 vs. ≤45 years), sex (male vs. female), T (T3–4 vs. T1–2) and N (N3–4 vs. N0–1) categories, and plasma EBV DNA (>2,000 vs. ≤2,000 copies/mL) are shown along with the corresponding Cox regression *P*-values. *A-IS*, active immune subtype; *CAF*, cancer-associated fibroblast; *EBV*, Epstein–Barr virus; *ECM*, extracellular matrix; *E-IS*, evaded immune subtype; *MDSC*, myeloid-derived suppressor cell; *non-IS*, non-immune subtype; *ssGSEA*, single-sample gene set enrichment analysis; *TAM*, tumour-associated macrophage

The top 50 overexpressed genes in the immune-enriched subtype versus non-IS were defined as an NPC immune-enriched signature, while those in the E-IS versus the A-IS were defined as an immune-evaded signature (Additional file 2: Table S5–6). We then applied the signatures to validation cohorts based on the NMF consensus to capture the immune subtypes. Clinical characteristics of patients in the validation cohorts are shown in Additional file 2: Table S7. In validation cohort 1, the molecular features of the immune subtypes were validated in accordance with the findings in the training cohort, demonstrating the reliability of the NPC immune subtypes (Fig. 2a). Survival analysis showed significantly better PFS for patients within A-IS than those within E-IS and non-IS (Fig. 2b). Patients within A-IS also had better overall survival and distant failure-free survival (Fig. 2c-d) compared to E-IS and non-IS. A trend of better locoregional failure-free survival was observed for A-IS than E-IS and non-IS (*P* = 0.099). It can be speculated that marginal significance is due to the excellent locoregional control of NPC [1].

To test the capacity of the immune subtypes to predict immunotherapy responses, we applied subclass mapping (SubMap) analysis to compare the gene expression profile of our immune subtypes with those of different groups of patients from two melanoma ICI cohorts. SubMap revealed that A-IS was genetically similar to melanoma tumours responding to PD-1 blockade (*P =* 0.012) and patients with long-term clinical benefit (Additional file 1: Figure S4), suggesting its predictive value.

**Fig. 3:**
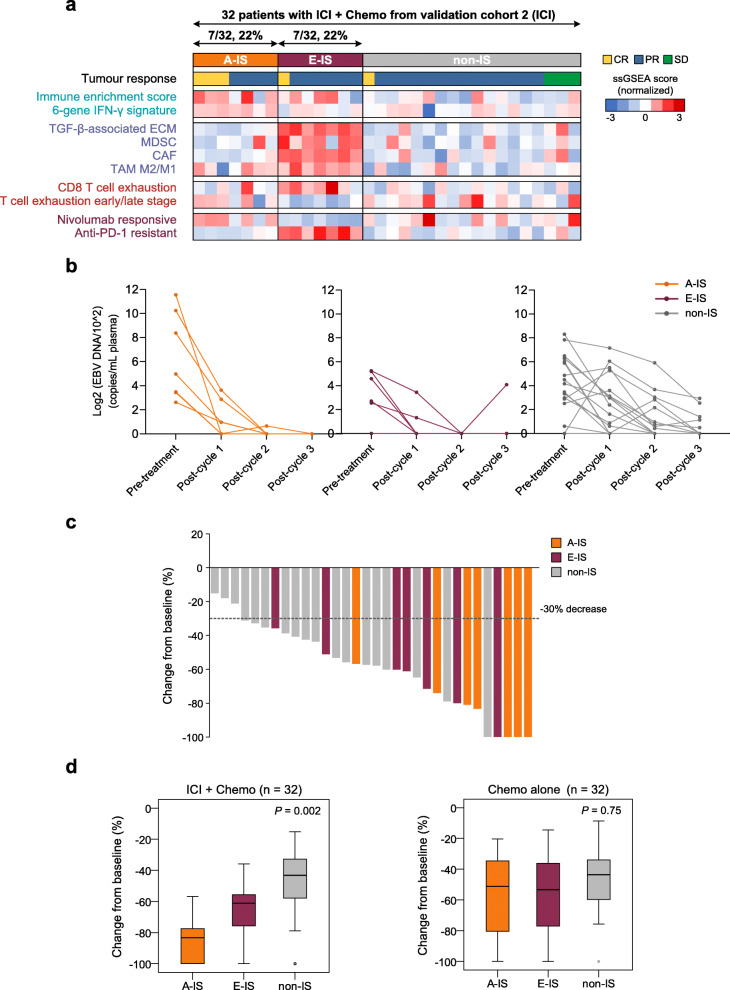
Verification of the immune subtypes in validation cohort 2 (ICI). **a** Heatmap representation of the tumour response and expression of immune-related signatures in A-IS, E-IS, and non-IS in 32 NPC patients receiving anti-PD-1 antibody combined with chemotherapy from validation cohort 2 (ICI). Tumour response was assessed after three cycles of ICI plus chemotherapy. In the heatmap, high and low ssGSEA scores are represented in red and blue, respectively. The presence and molecular characteristics of the immune subtypes were successfully validated. **b** Change in plasma EBV DNA levels in A-IS, E-IS, and non-IS during the treatment course in the 32 patients with ICI plus chemotherapy treatment. **c** Waterfall plot showing changes from baseline in the sum of longest target lesion diameters for each of the 32 patients with ICI plus chemotherapy treatment. PR was defined as a ≥30% decrease from baseline in the sum of diameters. **d** Box plots showing changes from baseline in the sum of longest target lesion diameters in A-IS, E-IS, and non-IS for the 32 patients receiving ICI plus chemotherapy (left) and the 32 matched patients receiving chemotherapy alone (right). The box plot centre corresponds to the median, with the box and whiskers corresponding to the interquartile range and 1.5× interquartile range, respectively. *P*-values were based on the Kruskal–Wallis rank-sum test. A significant P-value in the interaction test between ICI treatment (ICI plus chemotherapy versus chemotherapy alone) and the immune subtypes on tumour shrinkage was identified (*P* = 0.045). *A-IS*, active immune subtype; *CAF*, cancer-associated fibroblast; *Chemo*, chemotherapy; *CR*, complete response; *ECM*, extracellular matrix; *E-IS*, evaded immune subtype; *ICI*, immune checkpoint inhibitor; *MDSC*, myeloid-derived suppressor cell; *non-IS*, non-immune subtype; *PR*, partial response; *SD*, stable disease; *ssGSEA*, single-sample gene set enrichment analysis; *TAM*, tumour-associated macrophage

We next explored the predictive value of the immune subtypes in our validation cohort 2 (ICI) (*n* = 64). In the 32 NPC patients receiving three cycles of anti-PD-1 antibody in combination with chemotherapy from a prospective, multicenter study (NCT03025854), 14 patients (44%) were identified as A-IS (7/32, 22%) or E-IS (7/32, 22%) (Fig. 3a). Figure 3b illustrates the longitudinal plasma EBV DNA load during the treatment course. Although relatively higher pre-treatment EBV DNA levels were observed in patients within A-IS, all patients achieved a complete biological response (defined as undetectable EBV DNA in our previous study [10]) after treatment (Fig. 3b). In contrast, five of the 18 patients (5/18 28%) with non-IS still had detectable plasma EBV DNA after treatment. A decrease in target lesion size from baseline was observed in all 32 patients receiving ICI plus chemotherapy (Fig. 3c).

To avoid the potential bias caused by the chemotherapy in addition to ICI, validation cohort 2 also comprised 32 matched NPC patients receiving three cycles of chemotherapy alone. Significantly better tumour shrinkage was identified within A-IS for patients receiving ICI plus chemotherapy (*P* = 0.002). In contrast, no differences were identified among the three immune subtypes of the matched patients receiving chemotherapy alone (*P* = 0.75) (Fig. 3d). We further applied a treatment-by-covariate interaction test to examine the predictive ability of our immune subtypes and other potential ICI biomarkers in NPC [4]. Notably, we identified a significant interaction between treatment (ICI plus chemotherapy versus chemotherapy alone) and the immune subtypes on tumour shrinkage (*P* = 0.045), supporting the finding that effects of ICI treatment varied among different immune subtypes. In contrast, no interaction was observed for other biomarkers such as the IFN-γ signature (*P* = 0.85), *PD-L1* expression (*P* = 0.72), and anti-PD-1 resistant signature (*P* = 0.16). These results illustrated the beneficial association between A-IS and immunotherapy responses in NPC.

Understanding the intrinsic biology of these TME-based immune subtypes is critical for improving the efficacy of current immunotherapeutic strategies in NPC. For example, patients within A-IS may benefit from single-agent ICI or ICI combined with chemotherapy. For patients within E-IS, in addition to conventional chemotherapy, combination or sequential therapy with antibodies against PD-1, CTLA-4, and other immune checkpoints may improve clinical activity considering the late dysfunctional state [1]. Furthermore, patients within E-IS may obtain further benefit from inhibiting the immunosuppressive effects of TGF-β [11]. The efficacy of the TGF-β inhibitor, galunisertib, combined with nivolumab in advanced solid tumours is currently being investigated in a phase 1/2 trial (NCT02423343). For the remaining patients within non-IS, inducing a type I IFN response to attract T cell infiltration into the TME might be prioritized [12], and therapies targeting dysregulated cell cycle progression (e.g., palbociclib) may also be of interest. Still, it should be noted that the chemotherapy regimens were different in the ICI (GP regimen) and the matched (TPF regimen) cohorts, although these two regimens shared similar response rates in NPC in our previous trials [13, 14]. Besides, the ability of our immune subtypes to predict responses to different immunotherapeutic approaches and immune-related adverse events in different patient subgroups is worth exploring in larger cohorts with long-term outcomes. Our findings should be interpreted with these limitations in mind.

## Conclusions

Through evaluating the TME heterogeneity by virtual microdissection, we robustly classified the NPC TME into three biologically distinct immune subtypes. The distinctive biology of the immune subtypes further elucidates the differences in prognosis and immunotherapy responses. Our study would lay the foundation for future individualized immunotherapeutic strategies in NPC.

## Supplementary Information

**Additional file 1:** **Methods. Figure S1.** Study flow. HTA, Human Transcriptome Array; ICI, immune checkpoint inhibitor; NMF, non-negative matrix factorization; NPC, nasopharyngeal carcinoma; PSM, propensity score matching. **Figure S2.** Identification of an NMF immune factor. (**A**) We applied NMF (*k* = 5 factors or expression patterns) to analyze the gene expression profiles of nasopharyngeal carcinoma (NPC) samples in the training cohort (*n* = 113). One of the five factors (green bar) showed the highest ssGSEA scores in both immune enrichment score and 6-gene IFN-γ signature, as shown in the heatmap, indicating that it is an immune factor (or an immune expression pattern). High and low ssGSEA scores are represented in red and blue, respectively. (**B**) The top 100 exemplar immune factor genes characterized using DAVID confirmed immune-related functions. (**C**) NMF consensus-clustering of the training cohort using exemplar immune factor genes was refined by random forest classification. As shown in the heatmap, an immune-enriched subtype and a non-immune subtype. The ssGSEA scores of immune enrichment score and 6-gene IFN-γ signature are indicated; high and low scores are represented in red and blue, respectively. NMF, non-negative matrix factorization; NPC, nasopharyngeal carcinoma; ssGSEA, single-sample gene set enrichment analysis. **Figure S3.** Association of immune subtypes with tumoural genomic features and survival outcome. (**A**) Box plot showing similar number of non-synonymous mutations among the immune subtypes. (**B**) Box plot showing similar numbers of gene-level amplifications and deletions among the immune subtypes. (**C**) Box plot showing significantly higher cell cycling scores in non-IS. The box plot centre corresponds to the median, with the box and whiskers corresponding to the interquartile range and 1.5× interquartile range, respectively. *P*-values were based on the Kruskal–Wallis rank-sum test. (**D**) The proportion of CDKN2A deletions was significantly higher in non-IS. *P*-values were based on the Fisher’s exact test. (**E**) Kaplan–Meier curves for progression-free survival according to immune subtypes. A trend of better survival was observed for A-IS compared to E-IS and non-IS in 88 patients with available survival outcomes. P-values were calculated by log-rank test. A-IS, active immune subtype; CD4+ Tconv, conventional CD4+ T cells; CD8+ Tcyt, cytotoxic CD8+ T cells; CD8+ Tdys, dysfunctional CD8+ T cells; CD8+ Tnaï, naïve CD8+ T cells; DCs, dendritic cells; E-IS, evaded immune subtype; NK, natural killer; non-IS, non-immune subtype. **Figure S4.** Genetic similarity of the immune subtypes in different groups of patients from two melanoma ICI cohorts. (**A**) SubMap analysis of the immune subtypes in validation cohort 1 and four groups (anti-PD-1 responsive and non-responsive, and anti-CTLA-4 responsive and non-responsive) in melanoma ICI cohort 1. (**B**) SubMap analysis of the immune subtypes in validation cohort 1 and four groups (CR/PR/SD > 12 months, CR/PR/SD 6–12 months, CR/PR/SD <6 months, and PD for anti-PD-1 therapy) in melanoma ICI cohort 2. A-IS exhibited high similarity with anti-PD-1 responsive (*P* = 0.012), and CR/PR/SD >12 months for anti-PD-1 therapy (*P* = 0.036). A-IS, active immune subtype; CR, complete response; E-IS, evaded immune subtype; ICI, immune checkpoint inhibitor; non-IS, non-immune subtype; PD, progressive disease; PR, partial response; SD, stable disease. **Table S1.** Clinical cohorts used in this study. **Table S2.** Publicly available gene signatures used in this study. **Table S7.** Clinical characteristics of patients in the validation cohort 1 and validation cohort 2 (ICI).

**Additional file 2: Table S3.** GSEA results showing pathways enriched in the immune-enriched subtype vs. non-IS. **Table S4.** GSEA results showing pathways enriched in E-IS vs. A-IS. **Table S5.** List of top 50 genes over-expressed in the immune-enriched subtype vs. non-IS identified by Comparative Marker Selection (CMS). **Table S6.** List of top 50 genes over-expressed in E-IS vs. A-IS identified by Comparative Marker Selection (CMS).

## Data Availability

All raw and processed data were deposited in the European Genome-Phenome Archive (accession code: EGAS00001004542). The key processed and clinical data have been deposited in the Research Data Deposit public platform (www.researchdata.org.cn) (accession code RDDB2020000880) to validate the authenticity of this study.

## Abbreviations

A-IS: Active Immune Subtype
CMS: comparative marker selection
ECM: extracellular matrix
E-IS: Evaded Immune Subtype
HR: hazard ratio
ICI: immune checkpoint inhibitor
IMRT: intensity-modulated radiotherapy
NMF: non-negative matrix factorization
NPC: nasopharyngeal carcinoma
NTP: nearest template prediction
PD-1: programmed cell death protein 1
PD-L1: programmed cell death protein-ligand 1
PFS: progression-free survival
PSM: propensity score matching
SubMap: subclass mapping analysis
TME: tumour microenvironment
