# Supplementary material for: Unraveling tumour microenvironment heterogeneity in nasopharyngeal carcinoma identifies biologically distinct immune subtypes predicting prognosis and immunotherapy responses
Source: Mol Cancer. 2021 Jan 11;20:14. doi: 10.1186/s12943-020-01292-5 (PMC7798236; doi:10.1186/s12943-020-01292-5)
Supplement: Supplementary file 1 — Additional file 1: Methods. Figure S1. Study flow. HTA, Human Transcriptome Array; ICI, immune checkpoint inhibitor; NMF, non-negative matrix factorization; NPC, nasopharyngeal carcinoma; PSM, propensity score matching. Figure S2. Identification of an NMF immune factor. (A) We applied NMF (k = 5 factors or expression patterns) to analyze the gene expression profiles of nasopharyngeal carcinoma (NPC) samples in the training cohort (n = 113). One of the five factors (green bar) showed the highest ssGSEA scores in both immune enrichment score and 6-gene IFN-γ signature, as shown in the heatmap, indicating that it is an immune factor (or an immune expression pattern). High and low ssGSEA scores are represented in red and blue, respectively. (B) The top 100 exemplar immune factor genes characterized using DAVID confirmed immune-related functions. (C) NMF consensus-clustering of the training cohort using exemplar immune factor genes was refined by random forest classification. As shown in the heatmap, an immune-enriched subtype and a non-immune subtype. The ssGSEA scores of immune enrichment score and 6-gene IFN-γ signature are indicated; high and low scores are represented in red and blue, respectively. NMF, non-negative matrix factorization; NPC, nasopharyngeal carcinoma; ssGSEA, single-sample gene set enrichment analysis. Figure S3. Association of immune subtypes with tumoural genomic features and survival outcome. (A) Box plot showing similar number of non-synonymous mutations among the immune subtypes. (B) Box plot showing similar numbers of gene-level amplifications and deletions among the immune subtypes. (C) Box plot showing significantly higher cell cycling scores in non-IS. The box plot centre corresponds to the median, with the box and whiskers corresponding to the interquartile range and 1.5× interquartile range, respectively. P-values were based on the Kruskal–Wallis rank-sum test. (D) The proportion of CDKN2A deletions was significantly higher in non-IS. P-v [file 12943_2020_1292_MOESM1_ESM.docx]

**SUPPLEMENTARY MATERIALS AND METHODS**

**INDEX**

**METHODS.………….……………..………………………………….…………..……..….page 2**

**SUPPLEMENTARY FIGURES.………………………….…….………..…...…………...page 7**

**SUPPLEMENTARY TABLES.………………………………………….…....…...….….page 12**

**SUPPLEMENTARY REFERENCES.……………………...………….…....…..……….page 16**

**METHODS**

**Clinical specimens and study design**

We included 113 NPC samples profiled by RNA sequencing as the training cohort (**Figure S1**), which was previously described (GSE102349) [1]. The gene expression data were represented as fragments per kilobase per million (FPKM), log2-transformed, and filtered to include only genes expressed in ≥50% of the samples.

For the validation cohorts, pre-treatment paraffin-embedded biopsy tissue samples from NPC patients were profiled by microarray analysis (Affymetrix Human Transcriptome Array 2.0). Two pathologists (JPY and JZ) reevaluated all biopsy samples to confirm their eligibility (>70% tumour cells). In validation cohort 1, samples were retrospectively obtained from 150 patients treated between February 2010 and January 2016 at Sun Yat-sen University Cancer Center. All patients received concurrent cisplatin administered every 3 weeks for 2–3 cycles during IMRT, with or without induction chemotherapy using the TPF regimen (docetaxel plus cisplatin plus fluorouracil) [2] every 3 weeks for 2–4 cycles before IMRT. In validation cohort 2, 32 NPC patients receiving anti-PD-1 antibody from a prospective, multicenter study (ClinicalTrials.gov identifier NCT03025854) between January 2019 and December 2019 were included. These patients received three cycles of induction chemotherapy with the GP regimen (gemcitabine plus cisplatin every 3 weeks) [3], followed by two cycles of concurrent cisplatin along with IMRT; the anti-PD-1 antibody, sintilimab (200 mg every 3 weeks) [4], was administered in combination with chemotherapy during the treatment course. To avoid the potential bias caused by the additional chemotherapy to ICI, validation cohort 2 also comprised 32 matched NPC patients receiving three cycles of induction chemotherapy from validation cohort 1 for interaction test. As almost all NPC patients achieve a CR after radiotherapy [3], the tumour response was assessed after three cycles of induction chemotherapy with or without anti-PD-1 therapy. To assess treatment responses, changes from the baseline in the sum of the longest target lesion diameter per patient were calculated, and categorized as CR, PR, SD and PD, according to the Response Evaluation Criteria In Solid Tumours version 1.1 (RECIST 1.1) .

We also included two melanoma cohorts [5, 6] for SubMap analysis [7] with validation cohort 1 (**Figure S1**). The melanoma ICI cohort 1 consisted of 32 samples from melanoma patients receiving anti-CTLA-4 or anti-PD-1 therapy [5], while 103 melanoma tumours from patients receiving anti-PD-1 antibody were included in the melanoma ICI cohort 2 [6]. Details of all the cohorts in this study are summarized in **Table S1**.

Ethical approval was obtained from the institutional review board (SZR2019-069) in accordance with the Declaration of Helsinki; written informed consent was obtained from participants receiving ICI in validation cohort 2, while the requirement for informed consent was waived for participants in validation cohort 1 due to the retrospective analysis of anonymous data.

**Microarray data analysis**

For participants in the validation cohorts, total RNA was extracted from NPC tumour samples and hybridized to an Affymetrix HTA 2.0 microarray (Affymetrix, Santa Clara, CA, USA) according to the manufacturer’s instructions and using previously described experimental procedures [8]. In brief, total RNA was extracted using a QIAGEN FFPE RNeasy kit (QIAGEN GmbH, Germany), and the quantity and quality were analyzed using a NanoDrop 2000 spectrophotometer (ThermoFisher Scientific, Waltham, MA, USA) and an Agilent Bioanalyzer 2100 (Agilent Technologies, Santa Clara, USA). After amplification using an Ovation FFPE WTA System (NuGEN, San Carlos, CA, USA), an Encore Biotin Module (NuGEN) was used for fragmentation and labeling of total RNA. The total RNA was then hybridized to an Affymetrix Human Transcriptome Array 2.0 (Affymetrix) using a GeneChip Hybridization, Wash and Stain Kit (Affymetrix) following the manufacturer’s instructions. All slides were scanned with the GeneChip Scanner 3000 (Affymetrix). Raw data were obtained using Command Console Software 4.0 (Affymetrix) with the default setting, and were further preprocessed and normalized using Transcriptome Analysis Console version 4.0.1 (ThermoFisher Scientific).

**Classification and characterization of microenvironment-based immune subtypes**

We applied an NMF algorithm [9, 10] through GenePattern [11] for virtual analysis of the gene expression patterns of TME components in the training cohort. We selected k=5 as the number of NMF factors (or expression patterns), as it yielded a high cophenetic coefficient and effectively decomposed the cohort [9]. We then integrated the NMF factors with the gene signatures of the immune enrichment score and IFN-γ calculated by single-sample gene set enrichment analysis (ssGSEA, GenePattern module) to identify an NMF immune factor with high ssGSEA scores for both signatures (**Figure S2A**); the immune enrichment score reflects high immune cell abundance [12], while the 6-gene IFN-γ signature predicts clinical response to PD-1 blockade in other head and neck cancers [13]. The exemplar genes of the NMF immune factor were further characterized using DAVID (<https://david.ncifcrf.gov/>) to confirm their immune-related functions (**Figure S2B**). Subsequently, NMF consensus-clustering using the top 50 exemplar immune factor genes was refined by random forest classification [14] to reveal the immune-enriched subtype and non-IS in the training cohort (**Figure S2C**). The immune-enriched subtype was further subdivided into an E-IS and an A-IS using nearest template prediction (NTP) analysis with a signature identifying activated stromal responses [10]. Previously reported immune-related gene signatures (**Table S2**) calculated by ssGSEA were used to characterize different immune subtypes; we also assessed the distribution of diverse NPC immune cell subsets and cell cycling properties using their specific signatures identified in our single-cell RNA sequencing (scRNA-seq) study [15]. GSEA was used to identify pathways enriched in each subtype with a false discovery rate (FDR) <0.05, and differentially expressed genes between the immune subtypes were identified by comparative marker selection (CMS, GenePattern module).

**Validation of the immune subtypes in independent cohorts**

In the training cohorts, the top 50 overexpressed genes with the highest CMS scores in the immune-enriched subtype versus non-IS, and in E-IS versus A-IS were defined as the NPC immune-enriched and immune-evaded signatures, respectively. These signatures were applied to capture the immune subtypes based on NMF consensus in the validation cohorts. In brief, we first applied the immune-enriched signature to the whole cohort to identify the immune-enriched subtype versus non-IS. The immune-evaded signature was then applied to patients with the immune-enriched subtype to identify E-IS versus A-IS. The abovementioned immune-related gene signatures were used to characterize and validate the presence of the immune subtypes in validation cohorts.

SubMap analysis as aforementioned was performed through GenePattern; this bioinformatics method helps identify genetic similarity in gene expression profiles between subgroups from different independent cohorts [7]. We applied SubMap analysis to measure the similarity of NPC immune subtypes with different groups of patients (e.g., anti-PD-1 responsive and non-responsive) from the two melanoma ICI cohorts.

The matched NPC patients as aforementioned for interaction test were identified by PSM at a 1:1 ratio without replacement [16]. Propensity scores were determined by logistic regression based on the following baseline characteristics: age, sex, T and N categories, and plasma EBV DNA. As interaction would exist between two variables if the effect of one variable depends on the other [17], we conducted a test of treatment-by-covariate interaction using a linear regression model to identify potential interaction between ICI treatment (ICI plus chemotherapy versus chemotherapy alone) and different biomarkers (e.g., our immune subtypes, 6-gene IFN-γ signature [13]). Tumour shrinkage after treatment was selected as outcome variable, and a significant *P*-value in the interaction test indicated an association between the effects of ICI treatment and the biomarkers.

**Statistical analysis**

All statistical analyses were performed using R version 4.0.2 (<http://www.r-project.org>), GenePattern version 3.9 (<http://genepattern.broadinstitute.org>), and SPSS version 19.0 (IBM Corporation, Armonk, NY, USA). Correlations of immune subtypes with immune-related signatures and other assessed factors (e.g., tumour mutation burden, copy number alterations) were analyzed by Kruskal–Wallis rank-sum test (or Wilcoxon rank-sum test as indicated) and chi-squared test (or Fisher’s exact test as indicated) for continuous and categorical data, respectively. Treatment outcomes included PFS (calculated from the date of treatment to disease failure or death from any cause, whichever occurred first), overall survival (death from any cause), distant failure-free survival (distant failure or death from any cause) and locoregional failure-free survival (locoregional failure or death from any cause). Patients with a distant failure as a first event were censored for locoregional failure and vice versa. If both distant and locoregional failures occurred at the same time, patients were considered to have an event for both distant and locoregional failure-free survival. Kaplan–Meier estimates and log-rank tests were used for survival analysis of immune subtypes. We also uses a Cox proportional hazards model to test the independent significance of immune subtypes, with hazard ratios (HRs) and adjusted *P*-values obtained after correcting for age (>45 years vs. ≤45 years), sex (male vs. female), T (T3–4 vs. T1-2) and N (N3–4 vs. N0–1) categories, and plasma EBV DNA (>2,000 vs. ≤2,000 copies/mL). Two-tailed *P*-values<0.05 were considered to indicate statistical significance.

**SUPPLEMENTARY FIGURES**

**
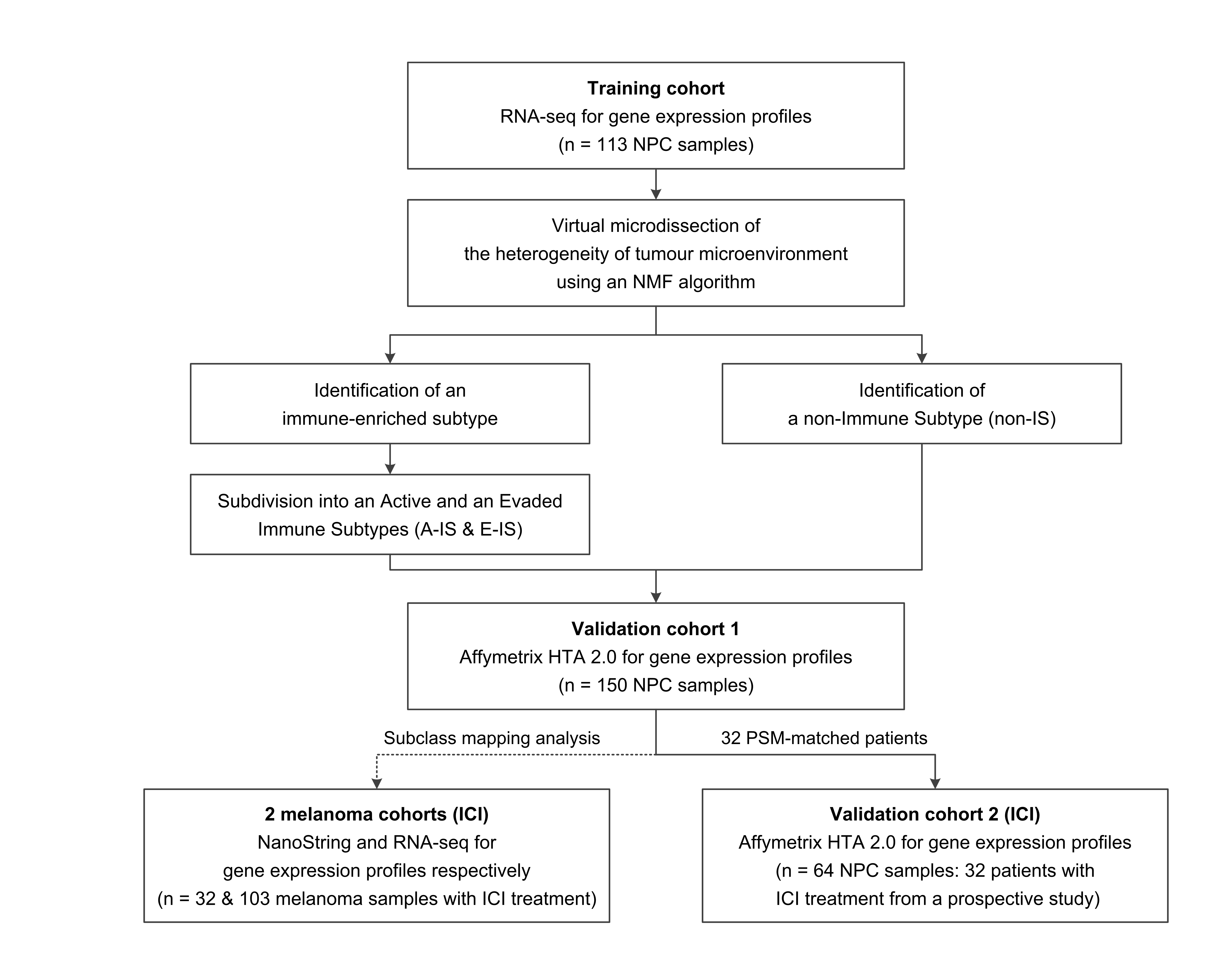
**

**Figure S1. Study flow.** HTA, Human Transcriptome Array; ICI, immune checkpoint inhibitor; NMF, non-negative matrix factorization; NPC, nasopharyngeal carcinoma; PSM, propensity score matching.

**
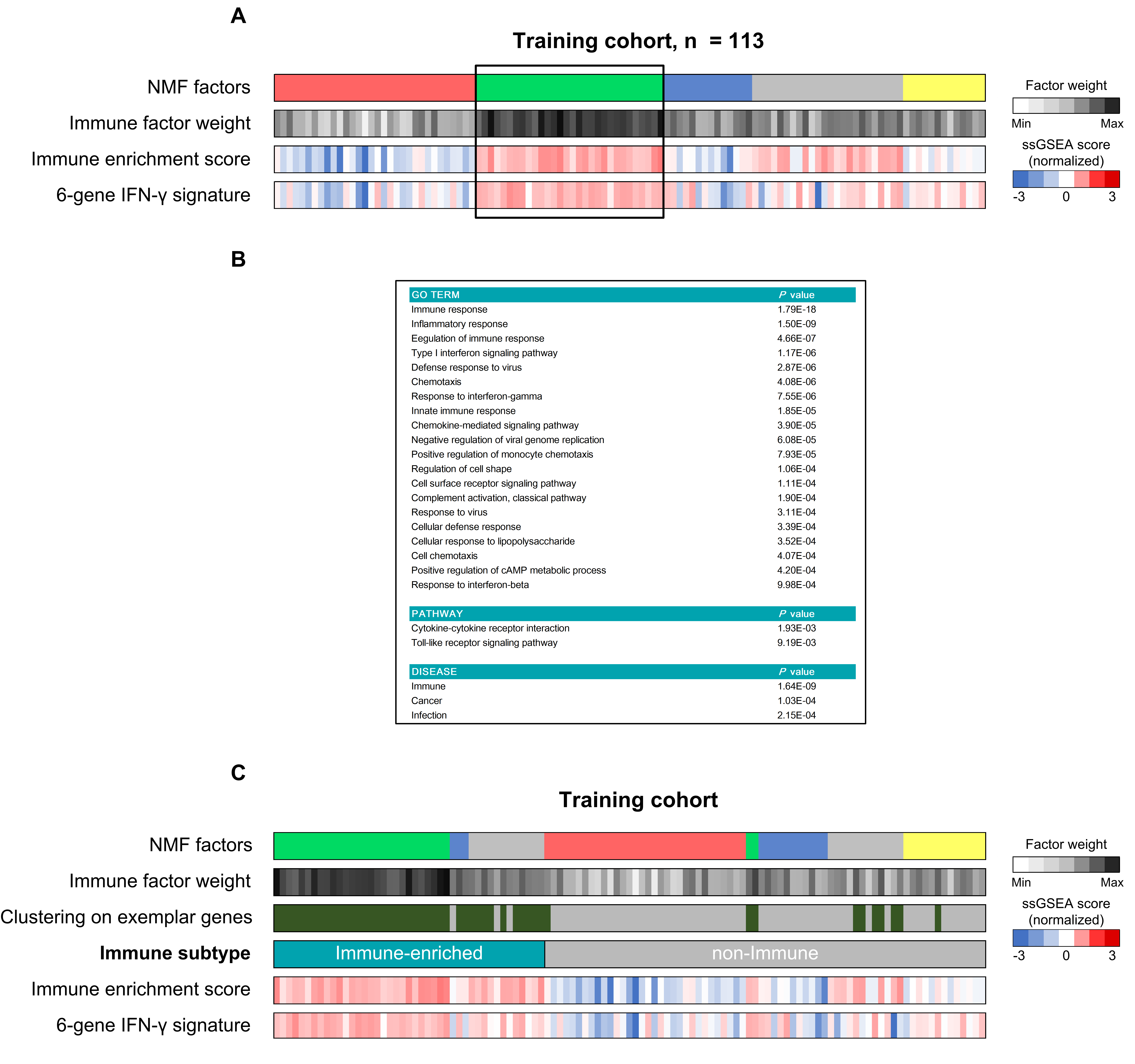
**

**Figure S2. Identification of an NMF immune factor.** **(A)** We applied NMF (k = 5 factors or expression patterns) to analyze the gene expression profiles of nasopharyngeal carcinoma (NPC) samples in the training cohort (n = 113). One of the five factors (green bar) showed the highest ssGSEA scores in both immune enrichment score and 6-gene IFN-γ signature, as shown in the heatmap, indicating that it is an immune factor (or an immune expression pattern). High and low ssGSEA scores are represented in red and blue, respectively. **(B)** The top 100 exemplar immune factor genes characterized using DAVID confirmed immune-related functions. **(C)** NMF consensus-clustering of the training cohort using exemplar immune factor genes was refined by random forest classification. As shown in the heatmap, an immune-enriched subtype and a non-immune subtype. The ssGSEA scores of immune enrichment score and 6-gene IFN-γ signature are indicated; high and low scores are represented in red and blue, respectively. NMF, non-negative matrix factorization; NPC, nasopharyngeal carcinoma; ssGSEA, single-sample gene set enrichment analysis.

**
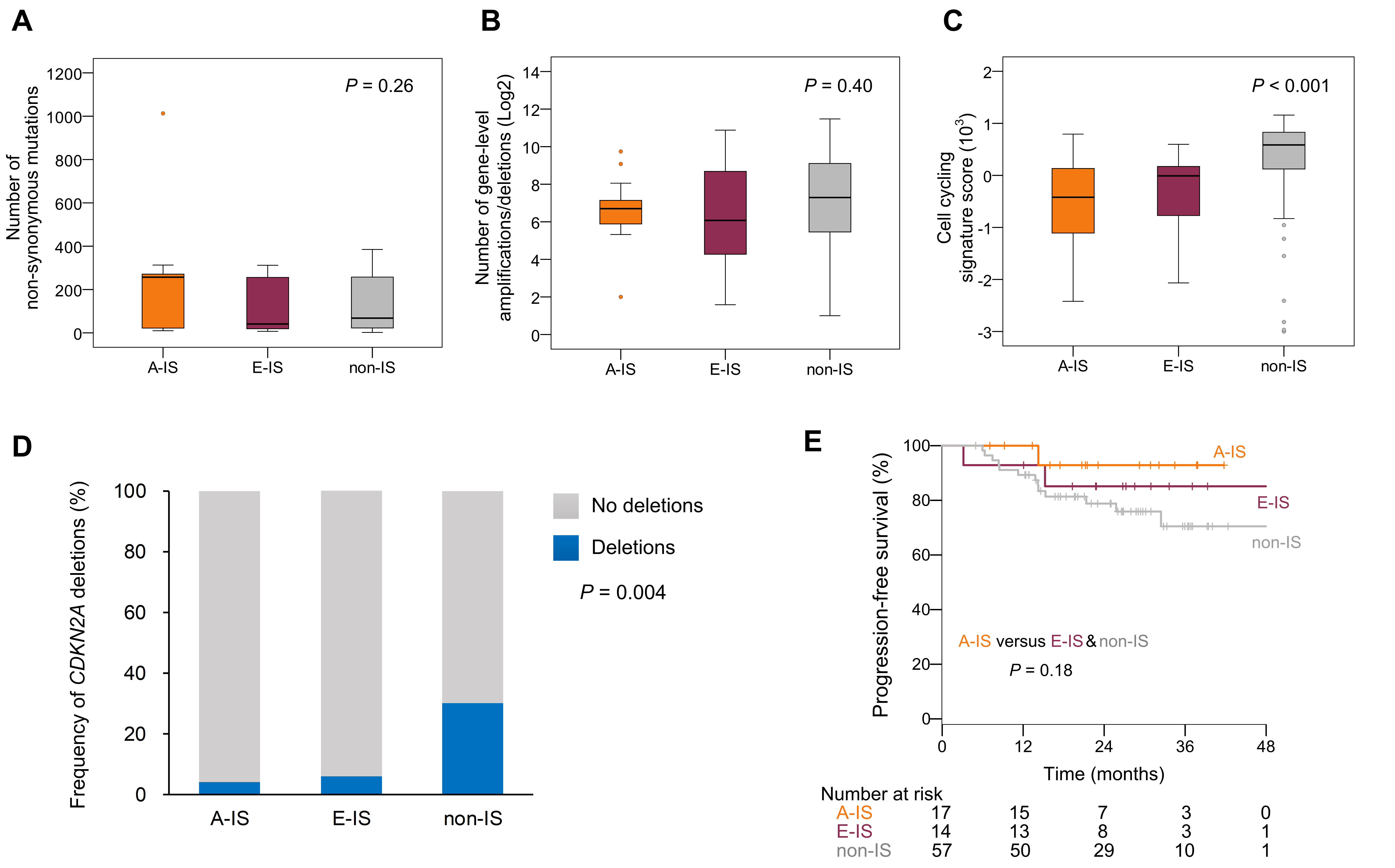
**

**Figure S3. Association of immune subtypes with tumoural genomic features and survival outcome. (A)** Box plot showing similar number of non-synonymous mutations among the immune subtypes. **(B)** Box plot showing similar numbers of gene-level amplifications and deletions among the immune subtypes. **(C)** Box plot showing significantly higher cell cycling scores in non-IS. The box plot centre corresponds to the median, with the box and whiskers corresponding to the interquartile range and 1.5× interquartile range, respectively. *P*-values were based on the Kruskal–Wallis rank-sum test. **(D)** The proportion of *CDKN2A* deletions was significantly higher in non-IS. *P*-values were based on the Fisher’s exact test. **(E)** Kaplan–Meier curves for progression-free survival according to immune subtypes. A trend of better survival was observed for A-IS compared to E-IS and non-IS in 88 patients with available survival outcomes. *P*-values were calculated by log-rank test. A-IS, active immune subtype; CD4^+^ T_conv_, conventional CD4^+^ T cells; CD8^+^ T_cyt_, cytotoxic CD8^+^ T cells; CD8^+^ T_dys_, dysfunctional CD8^+^ T cells; CD8^+^ T_naï_, naïve CD8^+^ T cells; DCs, dendritic cells; E-IS, evaded immune subtype; NK, natural killer; non-IS, non-immune subtype.

**
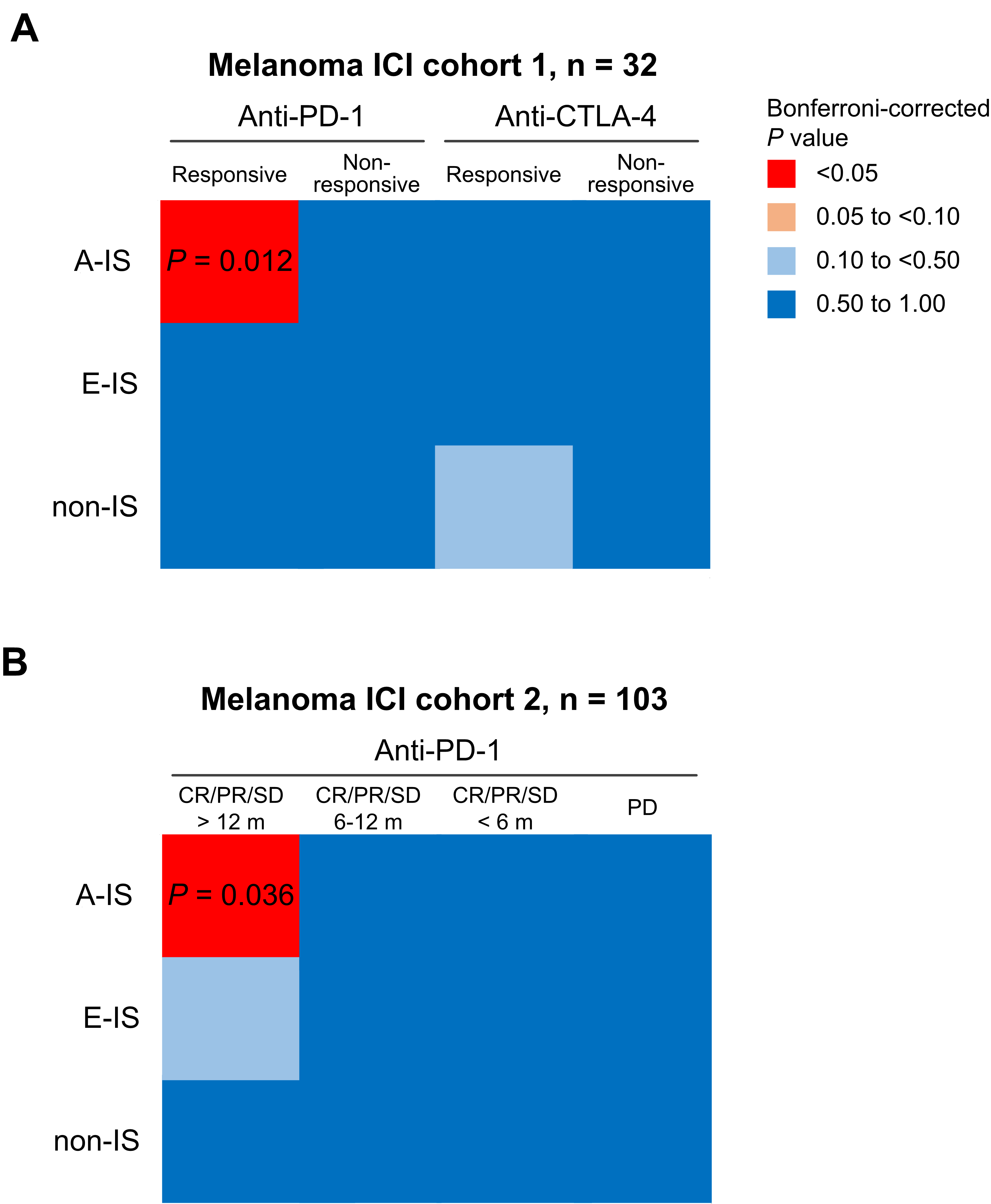
**

**Figure S4. Genetic similarity of the immune subtypes in different groups of patients from two melanoma ICI cohorts. (A)** SubMap analysis of the immune subtypes in validation cohort 1 and four groups (anti-PD-1 responsive and non-responsive, and anti-CTLA-4 responsive and non-responsive) in melanoma ICI cohort 1. **(B)** SubMap analysis of the immune subtypes in validation cohort 1 and four groups (CR/PR/SD > 12 months, CR/PR/SD 6–12 months, CR/PR/SD <6 months, and PD for anti-PD-1 therapy) in melanoma ICI cohort 2. A-IS exhibited high similarity with anti-PD-1 responsive (*P*=0.012), and CR/PR/SD >12 months for anti-PD-1 therapy (*P*=0.036). A-IS, active immune subtype; CR, complete response; E-IS, evaded immune subtype; ICI, immune checkpoint inhibitor; non-IS, non-immune subtype; PD, progressive disease; PR, partial response; SD, stable disease.

**SUPPLEMENTARY TABLES**

**Table S1. Clinical cohorts used in this study**

| **Cohort** | **Sample description** | **Data Array** | **Source** | **Identifier** |
| --- | --- | --- | --- | --- |
| Training cohort | 113 NPC samples | RNA-seq | GEO | GSE102349 |
| Validation cohort 1 | 150 NPC samples | Affymetrix Human Transcriptome Array 2.0 | This paper | -- |
| Validation cohort 2 (ICI) | 32 NPC samples | Affymetrix Human Transcriptome Array 2.0 | This paper | -- |
| Melanoma ICI cohort 1 | 32 Melanoma samples | NanoString nCounter system | Chen et al. Cancer Discov. 2016 | https://cancerdiscovery.aacrjournals.org/content/6/8/827.figures-only |
| Melanoma ICI cohort 2 | 103 Melanoma samples | RNA-seq | Jerby-Arnon et al. Cell. 2018 | https://portals.broadinstitute.org/single_cell/study/melanoma-immunotherapy-resistance |

Abbreviations: GEO, Gene Expression Omnibus; ICI, immune checkpoint inhibitor; NPC, nasopharyngeal carcinoma.

**Table S2. Publicly available gene signatures used in this study**

| **Signature name** | **Reference** | **Supplementary reference no.** |
| --- | --- | --- |
| Immune enrichment score | Yoshihara et al. Nat Commun. 2013 | [12] |
| 6-gene IFN-γ signature | Chow et al. J Clin Oncol. 2016 (suppl) | [13] |
| Activated stroma | Moffitt et al. Nat Genet. 2015 | [10] |
| Immune cell subsets | Cancer Genome Atlas Network. Cell. 2015 | [18] |
| T cells | Bindea et al. Immunity. 2013 | [19] |
| CD8 T cells | Bindea et al. Immunity. 2013 | [19] |
| T.NK. metagene | Alistar et al. Genome Med. 2014 | [20] |
| B-cell cluster | Iglesia et al. Clin Cancer Res. 2014 | [21] |
| Macrophages | Bindea et al. Immunity. 2013 | [19] |
| Cytotoxic cells | Bindea et al. Immunity. 2013 | [19] |
| Immunophenoscore | Charoentong et al. Cell Rep. 2017 | [22] |
| T cell-inflamed GEP | Cristescu et al. Science. 2018 | [23] |
| Expanded immune signature | Ayers et al. J Clin Invest. 2017 | [24] |
| TGF-β-associated ECM | Chakravarthy et al. Nat Commun. 2018 | [25] |
| MDSC | Yaddanapudi et al. Cancer Immunol Res. 2016 | [26] |
| CAF | Calon et al. Cancer Cell. 2012 | [27] |
| TAM M2/M1 | Beyer et al. PLoS One. 2012 | [28] |
| CD8 T cell exhaustion | Giordano et al. EMBO J. 2015 | [29] |
| T cell exhaustion early/late stage | Philip et al. Nature. 2017 | [30] |
| Nivolumab responsive | Riaz et al. Cell. 2017 | [31] |
| Anti-PD-1 resistant | Hugo et al. Cell. 2016 | [32] |

Abbreviations: CAF, cancer-associated fibroblast; ECM, extracellular matrix; GEP, gene expression profile; MDSC, myeloid-derived suppressor cell; TAM, tumor-associated macrophage.

**Table S7. Clinical characteristics of patients in the validation cohort 1 and validation cohort 2 (ICI)**

| **Variable** | **Validation cohort 1 (n = 150)** | | | |  | **Validation cohort 2 (ICI) (n = 32)** | | | |
| --- | --- | --- | --- | --- | --- | --- | --- | --- | --- |
|  | **Patients** | **A-IS**  **(n = 43)** | **E-IS**  **(n = 23)** | **non-IS**  **(n = 84)** |  | **Patients** | **A-IS**  **(n = 7)** | **E-IS**  **(n = 7)** | **non-IS**  **(n = 18)** |
| Age |  |  |  |  |  |  |  |  |  |
| ≤45 years | 89 (59%) | 26 (60%) | 8 (35%) | 55 (65%) |  | 20 (63%) | 3 (43%) | 6 (86%) | 11 (61%) |
| >45 years | 61 (41%) | 17 (40%) | 15 (65%) | 29 (35%) |  | 12 (38%) | 4 (57%) | 1 (14%) | 7 (39%) |
| Sex |  |  |  |  |  |  |  |  |  |
| Male | 111 (74%) | 35 (81%) | 17 (74%) | 59 (70%) |  | 23 (72%) | 4 (57%) | 4 (57%) | 15 (83%) |
| Female | 39 (26%) | 8 (19%) | 6 (26%) | 25 (30%) |  | 9 (28%) | 3 (43%) | 3 (43%) | 3 (17%) |
| T category^*^ |  |  |  |  |  |  |  |  |  |
| T1 | 5 (3%) | 2 (5%) | 2 (9%) | 1 (1%) |  | 0 (0%) | 0 (0%) | 0 (0%) | 0 (0%) |
| T2 | 23 (15%) | 7 (16%) | 1 (4%) | 15 (18%) |  | 3 (9%) | 1 (14%) | 1 (14%) | 1 (6%) |
| T3 | 77 (51%) | 24 (56%) | 10 (43%) | 43 (51%) |  | 14 (44%) | 5 (71%) | 3 (43%) | 6 (33%) |
| T4 | 45 (30%) | 10 (23%) | 10 (43%) | 25 (30%) |  | 15 (47%) | 1 (14%) | 3 (43%) | 11 (61%) |
| N category^*^ |  |  |  |  |  |  |  |  |  |
| N0 | 9 (6%) | 2 (5%) | 1 (4%) | 6 (7%) |  | 0 (0%) | 0 (0%) | 0 (0%) | 0 (0%) |
| N1 | 77 (51%) | 27 (63%) | 10 (43%) | 40 (48%) |  | 6 (19%) | 1 (14%) | 2 (29%) | 3 (17%) |
| N2 | 40 (27%) | 6 (14%) | 10 (43%) | 24 (29%) |  | 15 (47%) | 2 (29%) | 4 (57%) | 9 (50%) |
| N3 | 24 (16%) | 8 (19%) | 2 (9%) | 14 (17%) |  | 11 (34%) | 4 (57%) | 1 (14%) | 6 (33%) |
| Clinical stage^*^ |  |  |  |  |  |  |  |  |  |
| Stage II | 13 (9%) | 5 (12%) | 2 (9%) | 6 (7%) |  | 0 (0%) | 0 (0%) | 0 (0%) | 0 (0%) |
| Stage III | 75 (50%) | 21 (49%) | 11 (48%) | 43 (51%) |  | 9 (28%) | 2 (29%) | 4 (57%) | 3 (17%) |
| Stage IV | 62 (41%) | 17 (40%) | 10 (43%) | 35 (42%) |  | 23 (72%) | 5 (71%) | 3 (43%) | 15 (83%) |
| Plasma EBV DNA level |  |  |  |  |  |  |  |  |  |
| ≤2,000 copies/mL | 64 (43%) | 16 (37%) | 13 (57%) | 35 (42%) |  | 17 (53%) | 3 (43%) | 4 (57%) | 10 (56%) |
| >20,00 copies/mL | 86 (57%) | 27 (63%) | 10 (43%) | 49 (58%) |  | 15 (47%) | 4 (57%) | 3 (43%) | 8 (44%) |
| Induction chemotherapy |  |  |  |  |  |  |  |  |  |
| Yes | 99 (66%) | 28 (65%) | 18 (78%) | 53 (63%) |  | 32 (100%) | 7 (100%) | 7 (100%) | 18 (100%) |
| No | 51 (34%) | 15 (35%) | 5 (22%) | 31 (37%) |  | 0 (0%) | 0 (0%) | 0 (0%) | 0 (0%) |

Note: Data represent n (%) unless otherwise stated.

Abbreviations: A-IS, active immune subtype; EBV, Epstein–Barr virus; E-IS, evaded immune subtype; ICI, immune checkpoint inhibitor; non-IS, non-immune subtype.

^*^ According to the 8^th^ edition of the AJCC/UICC staging system.

SUPPLEMENTARY REFERENCES

1. Zhang L, MacIsaac KD, Zhou T, Huang PY, Xin C, Dobson JR, Yu K, Chiang DY, Fan Y, Pelletier M, et al: **Genomic Analysis of Nasopharyngeal Carcinoma Reveals TME-Based Subtypes.** *Mol Cancer Res* 2017, **15:**1722-1732.

2. Sun Y, Li WF, Chen NY, Zhang N, Hu GQ, Xie FY, Sun Y, Chen XZ, Li JG, Zhu XD, et al: **Induction chemotherapy plus concurrent chemoradiotherapy versus concurrent chemoradiotherapy alone in locoregionally advanced nasopharyngeal carcinoma: a phase 3, multicentre, randomised controlled trial.** *Lancet Oncol* 2016, **17:**1509-1520.

3. Zhang Y, Chen L, Hu GQ, Zhang N, Zhu XD, Yang KY, Jin F, Shi M, Chen YP, Hu WH, et al: **Gemcitabine and Cisplatin Induction Chemotherapy in Nasopharyngeal Carcinoma.** *N Engl J Med* 2019, **381:**1124-1135.

4. Gao S, Li N, Gao S, Xue Q, Ying J, Wang S, Tao X, Zhao J, Mao Y, Wang B, et al: **Neoadjuvant PD-1 inhibitor (Sintilimab) in NSCLC.** *J Thorac Oncol* 2020, **15:**816-826.

5. Chen PL, Roh W, Reuben A, Cooper ZA, Spencer CN, Prieto PA, Miller JP, Bassett RL, Gopalakrishnan V, Wani K, et al: **Analysis of Immune Signatures in Longitudinal Tumor Samples Yields Insight into Biomarkers of Response and Mechanisms of Resistance to Immune Checkpoint Blockade.** *Cancer Discov* 2016, **6:**827-837.

6. Jerby-Arnon L, Shah P, Cuoco MS, Rodman C, Su MJ, Melms JC, Leeson R, Kanodia A, Mei S, Lin JR, et al: **A Cancer Cell Program Promotes T Cell Exclusion and Resistance to Checkpoint Blockade.** *Cell* 2018, **175:**984-997 e924.

7. Hoshida Y, Brunet JP, Tamayo P, Golub TR, Mesirov JP: **Subclass mapping: identifying common subtypes in independent disease data sets.** *PLoS One* 2007, **2:**e1195.

8. Tang XR, Li YQ, Liang SB, Jiang W, Liu F, Ge WX, Tang LL, Mao YP, He QM, Yang XJ, et al: **Development and validation of a gene expression-based signature to predict distant metastasis in locoregionally advanced nasopharyngeal carcinoma: a retrospective, multicentre, cohort study.** *Lancet Oncol* 2018, **19:**382-393.

9. Brunet JP, Tamayo P, Golub TR, Mesirov JP: **Metagenes and molecular pattern discovery using matrix factorization.** *Proc Natl Acad Sci U S A* 2004, **101:**4164-4169.

10. Moffitt RA, Marayati R, Flate EL, Volmar KE, Loeza SG, Hoadley KA, Rashid NU, Williams LA, Eaton SC, Chung AH, et al: **Virtual microdissection identifies distinct tumor- and stroma-specific subtypes of pancreatic ductal adenocarcinoma.** *Nat Genet* 2015, **47:**1168-1178.

11. Reich M, Liefeld T, Gould J, Lerner J, Tamayo P, Mesirov JP: **GenePattern 2.0.** *Nat Genet* 2006, **38:**500-501.

12. Yoshihara K, Shahmoradgoli M, Martinez E, Vegesna R, Kim H, Torres-Garcia W, Trevino V, Shen H, Laird PW, Levine DA, et al: **Inferring tumour purity and stromal and immune cell admixture from expression data.** *Nat Commun* 2013, **4:**2612.

13. Chow LQM, Mehra R, Haddad RI, Mahipal A, Weiss J, Berger R, Eder JP, Burtness B, Tahara M, Keam B, et al: **Biomarkers and response to pembrolizumab (pembro) in recurrent/metastatic head and neck squamous cell carcinoma (R/M HNSCC).** 2016, **34:**6010-6010.

14. Sia D, Jiao Y, Martinez-Quetglas I, Kuchuk O, Villacorta-Martin C, Castro de Moura M, Putra J, Camprecios G, Bassaganyas L, Akers N, et al: **Identification of an Immune-specific Class of Hepatocellular Carcinoma, Based on Molecular Features.** *Gastroenterology* 2017, **153:**812-826.

15. Chen Y-P, Yin J-H, Li W-F, Li H-J, Chen D-P, Zhang C-J, Lv J-W, Wang Y-Q, Li X-M, Li J-Y, et al: **Single-cell transcriptomics reveals regulators underlying immune cell diversity and immune subtypes associated with prognosis in nasopharyngeal carcinoma.** *Cell Res* 2020.

16. Sturmer T, Joshi M, Glynn RJ, Avorn J, Rothman KJ, Schneeweiss S: **A review of the application of propensity score methods yielded increasing use, advantages in specific settings, but not substantially different estimates compared with conventional multivariable methods.** *J Clin Epidemiol* 2006, **59:**437-447.

17. Jiang P, Gu S, Pan D, Fu J, Sahu A, Hu X, Li Z, Traugh N, Bu X, Li B, et al: **Signatures of T cell dysfunction and exclusion predict cancer immunotherapy response.** *Nat Med* 2018, **24:**1550-1558.

18. Cancer Genome Atlas N: **Genomic Classification of Cutaneous Melanoma.** *Cell* 2015, **161:**1681-1696.

19. Bindea G, Mlecnik B, Tosolini M, Kirilovsky A, Waldner M, Obenauf AC, Angell H, Fredriksen T, Lafontaine L, Berger A, et al: **Spatiotemporal dynamics of intratumoral immune cells reveal the immune landscape in human cancer.** *Immunity* 2013, **39:**782-795.

20. Alistar A, Chou JW, Nagalla S, Black MA, D'Agostino R, Jr., Miller LD: **Dual roles for immune metagenes in breast cancer prognosis and therapy prediction.** *Genome Med* 2014, **6:**80.

21. Iglesia MD, Vincent BG, Parker JS, Hoadley KA, Carey LA, Perou CM, Serody JS: **Prognostic B-cell signatures using mRNA-seq in patients with subtype-specific breast and ovarian cancer.** *Clin Cancer Res* 2014, **20:**3818-3829.

22. Charoentong P, Finotello F, Angelova M, Mayer C, Efremova M, Rieder D, Hackl H, Trajanoski Z: **Pan-cancer Immunogenomic Analyses Reveal Genotype-Immunophenotype Relationships and Predictors of Response to Checkpoint Blockade.** *Cell Rep* 2017, **18:**248-262.

23. Cristescu R, Mogg R, Ayers M, Albright A, Murphy E, Yearley J, Sher X, Liu XQ, Lu H, Nebozhyn M, et al: **Pan-tumor genomic biomarkers for PD-1 checkpoint blockade-based immunotherapy.** *Science* 2018, **362**.

24. Ayers M, Lunceford J, Nebozhyn M, Murphy E, Loboda A, Kaufman DR, Albright A, Cheng JD, Kang SP, Shankaran V, et al: **IFN-gamma-related mRNA profile predicts clinical response to PD-1 blockade.** *J Clin Invest* 2017, **127:**2930-2940.

25. Chakravarthy A, Khan L, Bensler NP, Bose P, De Carvalho DD: **TGF-beta-associated extracellular matrix genes link cancer-associated fibroblasts to immune evasion and immunotherapy failure.** *Nat Commun* 2018, **9:**4692.

26. Yaddanapudi K, Rendon BE, Lamont G, Kim EJ, Al Rayyan N, Richie J, Albeituni S, Waigel S, Wise A, Mitchell RA: **MIF Is Necessary for Late-Stage Melanoma Patient MDSC Immune Suppression and Differentiation.** *Cancer Immunol Res* 2016, **4:**101-112.

27. Calon A, Espinet E, Palomo-Ponce S, Tauriello DV, Iglesias M, Cespedes MV, Sevillano M, Nadal C, Jung P, Zhang XH, et al: **Dependency of colorectal cancer on a TGF-beta-driven program in stromal cells for metastasis initiation.** *Cancer Cell* 2012, **22:**571-584.

28. Beyer M, Mallmann MR, Xue J, Staratschek-Jox A, Vorholt D, Krebs W, Sommer D, Sander J, Mertens C, Nino-Castro A, et al: **High-resolution transcriptome of human macrophages.** *PLoS One* 2012, **7:**e45466.

29. Giordano M, Henin C, Maurizio J, Imbratta C, Bourdely P, Buferne M, Baitsch L, Vanhille L, Sieweke MH, Speiser DE, et al: **Molecular profiling of CD8 T cells in autochthonous melanoma identifies Maf as driver of exhaustion.** *Embo J* 2015, **34:**2042-2058.

30. Philip M, Fairchild L, Sun L, Horste EL, Camara S, Shakiba M, Scott AC, Viale A, Lauer P, Merghoub T, et al: **Chromatin states define tumour-specific T cell dysfunction and reprogramming.** *Nature* 2017, **545:**452-456.

31. Riaz N, Havel JJ, Makarov V, Desrichard A, Urba WJ, Sims JS, Hodi FS, Martin-Algarra S, Mandal R, Sharfman WH, et al: **Tumor and Microenvironment Evolution during Immunotherapy with Nivolumab.** *Cell* 2017, **171:**934-949 e916.

32. Hugo W, Zaretsky JM, Sun L, Song C, Moreno BH, Hu-Lieskovan S, Berent-Maoz B, Pang J, Chmielowski B, Cherry G, et al: **Genomic and Transcriptomic Features of Response to Anti-PD-1 Therapy in Metastatic Melanoma.** *Cell* 2016, **165:**35-44.
